# Bisphenol A and ovarian cancer: emerging evidence and potential biological mechanisms

**DOI:** 10.3389/fcell.2026.1899695

**Published:** 2026-08-17

**Authors:** Lulu Zhang, Tong Yu, Zhongliu Bian, Tie Mao, Chengkui Xu

**Affiliations:** 1 Department of Hepatobiliary and Pancreatic Surgery, General Surgery Center, The First Hospital of Jilin University, Changchun, China; 2 First Operating Room, The First Hospital of Jilin University, Changchun, China; 3 Department of Cardiology Center, The First Hospital of Jilin University, Changchun, China

**Keywords:** bisphenol A, endocrine disruption, ovarian cancer, oxidative stress, tumor microenvironment

## Abstract

This review summarizes current evidence concerning the relationship between bisphenol A (BPA) exposure and ovarian cancer (OC). Human epidemiological studies have mainly examined possible associations between BPA concentrations and OC occurrence, but their cross-sectional or case–control designs do not establish that BPA exposure increases OC risk. Experimental studies in OC cell lines and tumor-bearing animals suggest that BPA may alter tumor-related phenotypes, including proliferation, migration, invasion, angiogenesis, and stemness. These findings concern potential tumor progression rather than patient prognosis. Separate studies in normal ovarian tissue and granulosa cells indicate possible ovarian toxicity, including oxidative stress, apoptosis, and steroidogenic disturbances, but these findings should not be interpreted as direct evidence of OC risk. Mechanistically, BPA exposure has been reported to be associated with changes in endocrine signaling, oxidative processes, metabolism, and epigenetic regulation in selected experimental models. Overall, evidence linking BPA exposure to ovarian-cancer-related outcomes remains limited, and current findings do not establish that BPA causes OC or worsens patient prognosis in humans.

## 1 Introduction

Ovarian cancer (OC) is a highly heterogeneous group of malignant tumors arising from the ovarian epithelium, germ cells, or stromal components. Based on histopathological characteristics, it is generally classified into epithelial ovarian cancer (EOC), germ cell tumors, and sex cord-stromal tumors, among which EOC is the most prevalent and the most aggressive subtype (Armstrong et al., 2021; Menon et al., 2018). Due to its insidious onset and the lack of effective early screening strategies, most patients are diagnosed at advanced stages, resulting in poor overall prognosis (Gaillard et al., 2025; Nash and Menon, 2020). In recent years, although advances in surgical techniques, platinum-based chemotherapy, and targeted therapies have improved the clinical management of OC, high recurrence rates and limited overall survival remain major challenges (Smolarz et al., 2025; Lheureux et al., 2019a). From a public health perspective, OC poses a substantial threat to women’s health and represents a considerable component of the global cancer burden (Li et al., 2025; Jayson et al., 2014). Current clinical approaches still face notable limitations in early detection and risk-reduction strategies, highlighting the need for improved risk assessment and more effective prevention and management approaches (Sessa et al., 2023; Lheureux et al., 2019b).

Bisphenol A (BPA) is a prototypical endocrine-disrupting chemical (EDC) extensively utilized in the manufacture of polycarbonate plastics and epoxy resins, which are widely present in food containers, beverage packaging, and medical devices (Abraham and Chakraborty, 2020; van den Brand et al., 2024; Murata and Kang, 2018). Structurally analogous compounds, including bisphenol S (BPS) and bisphenol F (BPF), have been increasingly introduced as substitutes, yet emerging evidence suggests that these analogs may exhibit comparable endocrine-disrupting activities (Rochester and Bolden, 2015). BPA can leach from consumer products, particularly under conditions of heat or repeated use, and enters the human body through multiple exposure routes, including dietary intake, dermal absorption, and inhalation (Michałowicz, 2014; Ďurovcová et al., 2022). Consequently, humans are subjected to chronic, low-dose exposure, typically within the nanomolar range. Following absorption, BPA undergoes metabolism; however, continuous environmental exposure results in sustained internal exposure. As an EDC, BPA has raised increasing concerns regarding its potential roles in human disease, particularly in hormone-related conditions including those affecting female reproductive health (Fenichel et al., 2013; Kozieł-Leszczyńska and Piastowska-Ciesielska, 2026; Khan et al., 2023). Experimental studies have demonstrated that even low-dose or prenatal exposure to BPA can disrupt the development and function of multiple organ systems, such as the brain, pancreas, and reproductive system (Fenichel et al., 2013; Trela-Kobędza and Ajduk, 2025; Isa et al., 2025). BPA has been the subject of extensive regulatory and toxicological evaluation. The Consortium Linking Academic and Regulatory Insights on the Toxicity of BPA (CLARITY-BPA) program, which integrated a standardized guideline-based study with academic investigations, was established to characterize the potential health effects of BPA across a broad range of exposure levels (Badding et al., 2025). In its 2023 re-evaluation, the European Food Safety Authority (EFSA) identified a public-health concern related to dietary BPA exposure and established a tolerable daily intake of 0.2 ng/kg body weight/day (Lambré et al., 2023). These assessments and related regulatory restrictions highlight the public-health relevance of BPA exposure; however, they do not demonstrate that BPA causes OC. BPA exposure has been investigated in relation to a broad spectrum of systemic outcomes, including endocrine disruption, metabolic disorders, cardiovascular effects, reproductive dysfunction, and cancer-related outcomes; however, the strength and causality of these associations vary substantially across conditions (Kozieł-Leszczyńska and Piastowska-Ciesielska, 2026; Sonavane and Gassman, 2019; Ma et al., 2019; Reyaz et al., 2025). Notably, human epidemiological studies have examined the possible association between BPA exposure and ovarian-cancer occurrence, whereas experimental studies have investigated cellular phenotypes relevant to tumor progression. These types of evidence address distinct outcomes and should not be interpreted as equivalent (Li et al., 2026; Li and Li, 2026; Arcieri et al., 2026). Therefore, a comprehensive understanding of the link between BPA and OC is of significant importance. In this review, we distinguish human evidence concerning ovarian-cancer risk from experimental evidence related to tumor progression and from studies examining general ovarian or granulosa-cell toxicity. Evidence regarding ovarian-cancer prognosis is considered separately because it remains limited.

## 2 Literature search and selection methodology

A literature search was conducted in PubMed and Web of Science, covering the period from database inception through May 2026. The search strategy combined terms related to bisphenol A (BPA), ovarian cancer (OC), exposure, and carcinogenic mechanisms. Search terms included “bisphenol A,” BPA, “bisphenol analogues,” BPS, and BPF, combined with “ovarian cancer,” “ovarian carcinoma,” or “ovarian neoplasm.” Where appropriate, additional terms were used, including “endocrine disruption,” “oxidative stress,” “proliferation,” “migration,” “angiogenesis,” “epithelial–mesenchymal transition,” “epigenetic,” “PI3K/Akt,” “MAPK/ERK,” and “JAK/STAT3.” Studies were eligible for inclusion if they (1) investigated exposure to BPA or related bisphenols; (2) examined OC risk, development, progression, prognosis, or relevant biological mechanisms; and (3) provided epidemiological, experimental, animal, mechanistic, or integrative evidence. Reviews, editorials, conference abstracts lacking sufficient data, and studies unrelated to OC or bisphenol exposure were excluded. Only studies published in English were included. As this was a narrative review, the included studies were not quantitatively pooled, and no formal meta-analysis was performed. The quality and limitations of the available evidence were assessed qualitatively, taking into account study design, sample size, exposure assessment, adjustment for potential confounders, outcome measurement, experimental controls, the biological relevance of exposure concentrations, reproducibility, and consistency across studies. Causal inferences were interpreted cautiously, particularly for cross-sectional and case–control studies.

## 3 Evidence linking BPA exposure to OC risk

Current evidence from epidemiological studies, integrative analyses, and experimental models suggests a possible association between BPA exposure and OC, although these findings should not be interpreted as equivalent evidence of OC risk, prognosis, tumor progression, or general ovarian toxicity (Table 1). At the population level, several case–control and cross-sectional studies have reported higher urinary BPA concentrations in women with OC than in controls. However, these studies cannot establish whether BPA exposure preceded disease development. For example, a cross-sectional study of 50 women, including 24 with epithelial OC or borderline ovarian tumors (EBOTs) and 26 controls, reported higher urinary BPA levels in patients (p < 0.001), as measured by GC–MS/MS (Sladič et al., 2025). The small sample size, single urine measurement, and potential effects of urinary dilution, renal function, and short-term day-to-day variation in BPA excretion limit the interpretation of this finding. Similarly, a case–control study of 30 OC patients and 30 controls reported higher urinary BPA levels, together with increased ROS and KRT4 expression and reduced SOD activity (p < 0.001) (Alsaeed et al., 2026). These results may reflect associations with disease status or related lifestyle and clinical factors, and residual confounding and reverse causation cannot be excluded. Moreover, ROS, SOD, and KRT4 findings do not demonstrate increased OC risk or poorer patient prognosis. Where available, effect estimates with 95% confidence intervals should be reported rather than relying mainly on p values. A recent integrative study combining NHANES data with genetic and bioinformatics analyses found higher urinary BPA levels in 66 OC cases than in 291 controls (p < 0.05). In the MR analysis, BPA-related genetic variants were selected from the genome-wide association studies (GWAS) Catalog (GCST90308719, N = 652) using a threshold of p < 1 × 10^−5^, followed by LD clumping. Twelve independent single-nucleotide polymorphisms (SNPs) with F > 10 (mean F = 15.3) was retained as instruments. The IVW analysis showed a positive association between genetically predicted BPA exposure and OC risk (OR = 1.25, 95% CI: 1.02–1.54, p = 0.029). Cochran’s Q test (p = 0.9989) and the MR-Egger intercept test (p = 0.59) indicated no substantial heterogeneity or horizontal pleiotropy, and leave-one-out analyses supported the robustness of the estimate. However, the study did not clearly report the ancestry of the GWAS populations or a formal directionality assessment. Therefore, these findings should be considered suggestive rather than definitive evidence of causality and require independent replication. The SKOV3-cell findings of increased proliferation, migration, and invasion represent experimental cellular phenotypes and do not directly demonstrate tumor progression or poorer patient outcomes (Luo et al., 2026). Collectively, these studies suggest differences in BPA exposure or BPA-related responses among women with OC, but they do not establish that BPA increases OC risk, affects prognosis, or causes the disease.

**TABLE 1:** Summary of key evidence regarding bisphenols and OC.

Study	Population/model	Chemical	Exposure concentration/dose and biomarker	Main result	Principal limitation
Sladič et al. (2025)	Women with EOC/EBOT and healthy controls	BPA	Total urinary BPA measured by GC–MS/MS	Urinary BPA was higher in women with EOC/EBOT and associated with urinary creatinine; no association with serum AMH was observed	Small observational study; BPA was measured after diagnosis, preventing causal inference
Alsaeed et al. (2026)	30 patients and 30 controls	BPA	Total urinary BPA, ROS, SOD, and KRT4 expression	Patients had higher BPA, ROS, and KRT4 expression and lower SOD activity	Small case–control study; possible selection and residual confounding; post-diagnostic exposure measurement
Luo et al. (2026)	NHANES analysis, MR, omics, and SKOV3 experiments	BPA	Total urinary BPA; MR; integrated omics; cell assays	BPA was associated with OC, and experimental data indicated increased proliferation, migration, and invasion	Cross-sectional human data, possible limitations of MR assumptions, and reliance on one cell line
Sang et al. (2021)	OVCAR-3 cells	BPA	1–100 nM; *in vitro* exposure for 24 h or 5 days	BPA promoted proliferation, migration, invasion, and adhesion through ERα-dependent signaling	*In vitro* study using one cell line; does not establish tumor initiation in humans
Shi et al. (2017)	OVCAR-3 cells	BPA	10–12 –10–7 mol/L; *in vitro* exposure for 24 h	BPA increased proliferation, glycolysis, ATP, lactate, and pyruvate through ERα signaling	*In vitro* model; exposure conditions may not fully represent human tissue exposure
Kim et al. (2015)	BG-1 cells	BPA and nonylphenol	1 μM,*in vitro* exposure for 6–96 h	BPA promoted EMT markers and cell migration through an ER-dependent pathway	Cell-line study; included nonylphenol, limiting BPA-specific interpretation
Yuan et al. (2023)	Ovarian-cancer cells and mice	BPA and BPS	0–1000 nM *in vitro*; 100 nM showed marked effects; mouse exposure	BPA and BPS increased cancer-stemness markers and metastasis through PINK1/p53-related mitophagy	Limited information on human relevance; cell and animal models may not reflect clinical exposure
Guo et al. (2020)	OVCAR-3 cells	BPA	0.1 μM,*in vitro* exposure for 24 or 72 h	BPA increased cancer-stem-cell spheres, CD44^high^CD24^low^ cells, and CSC markers	*In vitro* study; no direct evidence of increased OC incidence in humans.
Hassan et al. (2024)	Female albino rats	BPA with γ-radiation	BPA, 50 mg/kg; radiation, 2–6 Gy	BPA and radiation altered ovarian toxicity, estrogen-receptor signaling, and PI3K/Akt-related pathways	High experimental exposure; combined radiation limits attribution to BPA alone; no established ovarian tumors.
Park and Choi (2014)	BG-1 cells and xenograft mice	BPA and other EDCs	1 μM; *in vitro* exposure for 15 min–2h; 150 mg/kg body weight every 2 days for 8 days	BPA inhibited TGF-β/Smad3 signaling and promoted growth-related changes	Mixed exposure model; subtype and human-dose relevance remain uncertain
Rajaura et al. (2024)	Swiss mice	BPA	1 or 5 mg/kg, alternate days for 4 months	BPA increased ROS, inflammatory changes, and selected ovarian CSC populations	Relatively high exposure; ovarian CSC changes do not demonstrate spontaneous OC development
Bhardwaj et al. (2025)	Female mice	BPA	1 or 5 mg/kg, alternate days for 4 months	BPA altered ovarian epithelial-cell populations, apoptosis, inflammation, antioxidant responses, and tumor-suppressor genes	Animal study with high doses; pathological changes cannot be directly extrapolated to human OC

The BG-1 cells used in the two studies were obtained from Dr. K. S. Korach at the National Institute of Environmental Health Sciences, NIH. The original studies did not report STR authentication, *mycoplasma* testing, or passage-history verification. Published concerns regarding the identity of one widely used BG-1 variant and its relationship to the MCF-7 breast-cancer cell line should therefore be considered when interpreting these findings.

Abbreviations OC, ovarian cancer; EOC, epithelial ovarian cancer; EBOT, epithelial borderline ovarian tumor(s); BPA, bisphenol A; BPS, bisphenol S; BPF, bisphenol F; GC–MS/MS, gas chromatography–tandem mass spectrometry; AMH, anti-Müllerian hormone; ROS, reactive oxygen species; SOD, superoxide dismutase; KRT4, keratin 4; NHANES, National Health and Nutrition Examination Survey; MR, Mendelian randomization; ERα, estrogen receptor alpha; EMT, epithelial–mesenchymal transition; CSC, cancer stem cell; CD, cluster of differentiation; PINK1, PTEN-induced putative kinase 1; p53, tumor protein p53; TGF-β, transforming growth factor beta; EDCs, endocrine-disrupting chemicals; PI3K, phosphoinositide 3-kinase; Akt, protein kinase B; SKOV3 and OVCAR-3, human ovarian cancer cell lines; γ-radiation, gamma radiation.

At the cellular level, experimental studies suggest that BPA may induce several phenotypic changes related to OC progression. However, these findings represent effects observed in cancer-cell or animal models and should not be interpreted as direct evidence of increased OC risk, poorer patient prognosis, or clinical tumor progression. BPA has been reported to enhance OC cell proliferation, including at relatively low concentrations. In OVCAR-3 cells, this effect was accompanied by increased glycolysis-related metabolic activity, ATP production, lactate levels, and pyruvate levels, and was mediated, at least in part, through estrogen receptor-α (ERα) signaling (Sang et al., 2021; Shi et al., 2017). These findings indicate that BPA can affect the growth and metabolism of established OC cells, but they do not demonstrate that BPA initiates OC in humans. In addition to its effects on proliferation, BPA exposure has been associated with increased migration, invasion, and adhesion of OC cells. These changes were accompanied by increased expression of migration- and invasion-related markers, such as MMP-2 and MMP-9, as well as EMT-related alterations, including increased vimentin, snail, and slug expression and reduced E-cadherin expression (Sang et al., 2021; Kim et al., 2015). Such findings support the induction of a more motile and invasive cellular phenotype *in vitro*. Nevertheless, they should not be considered equivalent to evidence of metastasis or tumor progression in patients, and the effects of BPA on clinical outcomes such as recurrence, survival, or treatment response remain unclear. Importantly, BPA has also been reported to enhance cancer stem cell–like properties in OC cells. This was reflected by increased sphere-forming ability, expansion of the CD44^high^CD24^low^ subpopulation, and upregulation of stemness-associated markers, including OCT4, NANOG, SOX2, ALDH1A1, and CD133 (Yuan et al., 2023; Guo et al., 2020). These changes may indicate increased cellular plasticity and may be relevant to tumor aggressiveness, metastasis, or treatment resistance; however, their significance for patient prognosis has not been established. Moreover, the reported effects were obtained under specific experimental conditions, and their relevance to human exposure levels requires further validation. Separately, studies of ovarian granulosa cells have reported that BPA exposure can alter cellular proliferation, reduce cell viability, disrupt steroidogenesis and gene expression, and induce oxidative stress, autophagy, and apoptosis (Celar Sturm and Virant-Klun, 2023). These findings reflect general ovarian or granulosa-cell toxicity and should be distinguished from evidence concerning OC risk, prognosis, or tumor progression. Overall, BPA exposure is associated with several tumor-related cellular phenotypes in OC models, including enhanced proliferation, migration, invasion, and stem cell–like characteristics. However, current experimental evidence does not establish that BPA causes OC, promotes clinical metastasis, or worsens the prognosis of patients with OC.

At the organismal level, direct animal evidence linking BPA exposure to OC risk remains limited. Available studies mainly indicate ovarian and reproductive toxicity, including endocrine disruption, altered ovarian morphology, epithelial hyperplasia, inflammatory infiltration, oxidative stress, and changes in antioxidant responses (Hassan et al., 2024). These findings demonstrate adverse effects on ovarian tissue but should not be considered equivalent to evidence of ovarian-cancer development or patient prognosis. In tumor-bearing models, BPA exposure has also been associated with tumor-related effects. In BG-1 xenograft mice, BPA exposure was associated with increased tumor growth and was accompanied by changes in tumor-cell proliferation and TGF-β/Smad3-related signaling (Park and Choi, 2014). However, xenograft models involving transplanted cancer cells cannot determine whether BPA initiates OC *in vivo*, and they do not directly establish effects on metastasis, survival, recurrence, or treatment response in patients. In addition, no direct evidence currently demonstrates that BPA alters chemotherapy sensitivity or causes chemotherapy resistance in ovarian-cancer models. Moreover, BPA exposure in mice has been associated with increased oxidative stress, reduced antioxidant gene expression, inflammatory changes, and an increased proportion of ovarian cells with cancer stem cell–like markers (Rajaura et al., 2024). These findings may indicate a tissue environment conducive to abnormal cellular responses, but they do not by themselves demonstrate tumor initiation or clinical tumor progression. Prolonged exposure has also been reported to induce borderline epithelial changes, altered epithelial-cell composition, apoptosis or necrosis, and changes in inflammatory, antioxidant, and tumor-suppressor pathways in ovarian tissue (Bhardwaj et al., 2025). Such results should be interpreted as evidence of ovarian tissue toxicity and early tumor-related alterations rather than definitive proof of ovarian carcinogenesis. Complementary integrative analyses have identified BPA-associated gene signatures related to OC and malignant epithelial-cell phenotypes (Luo et al., 2026); however, these findings are mechanistic or associative and require validation in independent *in vivo* models. Overall, current evidence suggests that BPA exposure can produce ovarian toxicity and may enhance tumor-related phenotypes in certain experimental models, but direct evidence that BPA causes OC, promotes clinical tumor progression, or worsens patient prognosis remains insufficient.

Importantly, evidence concerning BPA substitutes, including BPS and BPF, should be considered separately from evidence concerning BPA itself. In a nested case–control study including 318 patients with advanced high-grade serous OC, higher urinary BPS concentrations were significantly associated with poorer survival outcomes, with an odds ratio of 2.18 for the highest versus lowest exposure groups. A dose–response relationship was also observed, as each standard deviation increase in urinary BPS concentration was associated with reduced survival (OR = 1.36) (Li et al., 2026). These findings suggest that BPS exposure may be associated with tumor progression or prognosis among patients with established OC. However, this study did not evaluate BPA exposure, did not assess OC initiation, and therefore should not be interpreted as direct evidence that BPA or BPS causes OC. Evidence concerning BPF and other BPA substitutes remains limited and requires separate evaluation (Edaes and de Souza, 2022).

**FIGURE 1:**
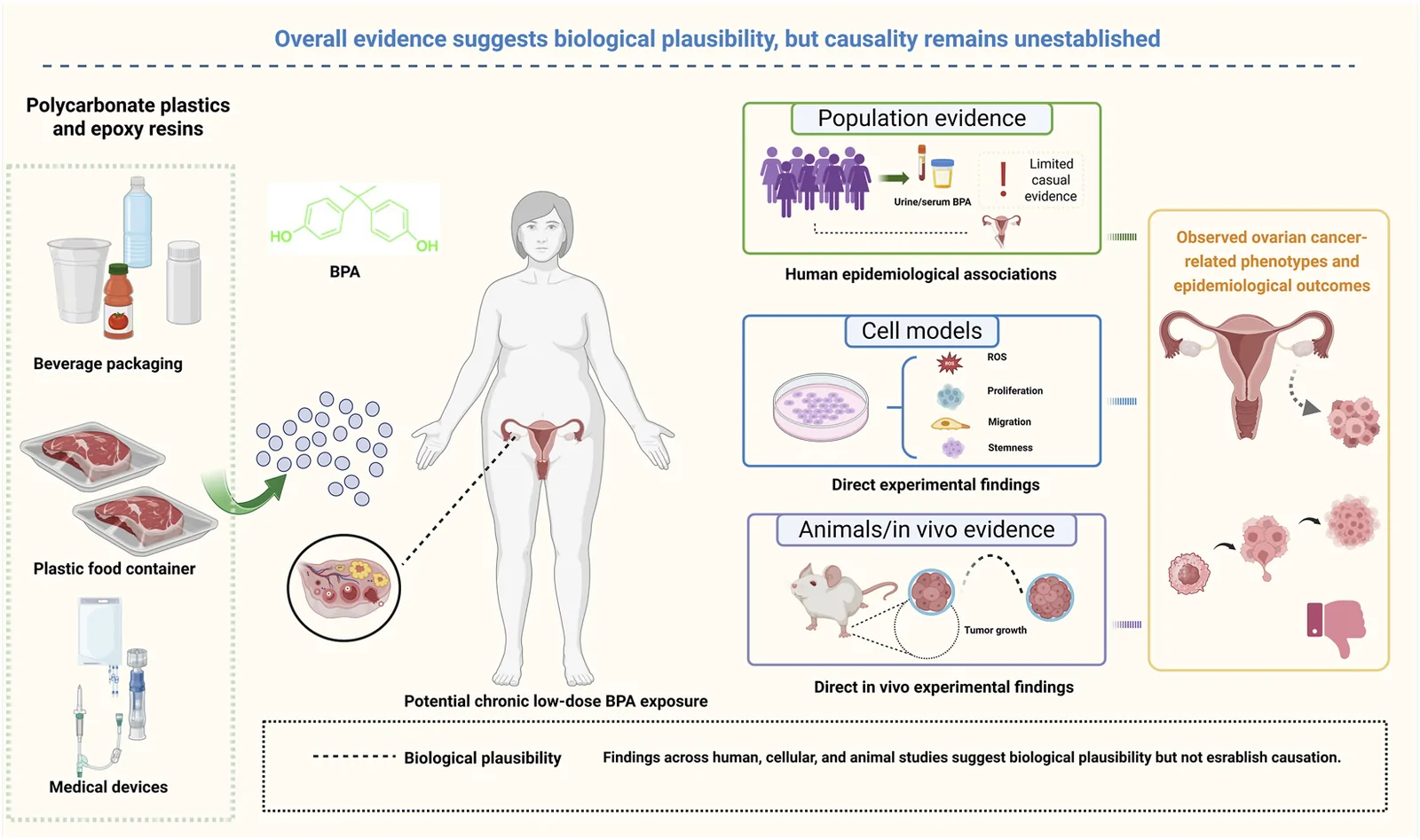
Overview of the available evidence linking BPA exposure to ovarian cancer-related outcomes. Potential sources of BPA exposure include beverage packaging, plastic food containers, and medical devices. Human epidemiological associations, direct findings from cell models, and direct *in vivo* experimental findings are presented as distinct evidence categories. The reported outcomes include ovarian cancer-related phenotypes and epidemiological observations. Collectively, these findings support biological plausibility but do not establish a confirmed causal relationship between BPA exposure and ovarian cancer. All connections are schematic and do not imply a proven causal sequence. Created with BioRender.com.

Taken together, evidence from population studies, cellular experiments, and animal models provides biological plausibility for a possible association between BPA exposure and OC-related outcomes. However, these findings should not be interpreted as evidence that BPA causes OC in humans (Figure 1).

## 4 Potential biological pathways associated with BPA exposure and ovarian-cancer-related phenotypes

Evidence summarized in this section was classified according to the type of evidence available. Direct experimental evidence includes findings from BPA-exposed ovarian cells, normal ovarian tissues, and animal models, such as changes in cell proliferation, migration, invasion, signaling proteins, and tumor-related phenotypes. In contrast, pathway-enrichment analyses, molecular-docking simulations, The Cancer Genome Atlas (TCGA) correlations, Mendelian randomization, network toxicology, and immune-cell deconvolution provide computational, statistical, or indirect evidence. These approaches are hypothesis-generating and can suggest potential targets or pathways, but they do not demonstrate that BPA directly activates a specific pathway or causes OC through a confirmed mechanism.

### 4.1 Endocrine disruption and hormone receptor signaling

A potential biological pathway linking BPA exposure to OC-related cellular phenotypes involves its estrogen-mimicking activity and the consequent modulation of hormone-receptor signaling. Due to its structural similarity to endogenous estrogens, BPA has been reported to interact with estrogen receptor alpha (ERα) and may modulate estrogen receptor-related signaling (Hwang et al., 2011). These signaling changes may be associated with altered proliferative activity in selected experimental models. In the genomic pathway, activated ERα translocates to the nucleus and binds estrogen response elements (EREs), inducing transcription of key cell cycle regulators such as CCND1 (Cyclin D1), thereby accelerating the G1/S transition. This effect is further amplified by crosstalk between ERα and insulin-like growth factor-1 receptor (IGF-1R) signaling, which activates the IRS-1/Akt axis and reinforces Cyclin D1 expression (Kang et al., 2013; Hwang et al., 2013a; Hwang et al., 2013b; Hall and Korach, 2013). In parallel, BPA exposure promotes upregulation of additional cell cycle drivers, including cyclin-dependent kinases (CDKs) and MYC, while suppressing inhibitory regulators such as p21 and enhancing CDK2 activity, collectively favoring continuous cell division (Kang et al., 2012). Concurrently, non-genomic ESR1 signaling rapidly activates major oncogenic cascades, particularly phosphoinositide 3-kinase/protein kinase B (PI3K/Akt), mitogen-activated protein kinase/extracellular signal-regulated kinase (MAPK/ERK), and Janus kinase/signal transducer and activator of transcription 3 (JAK/STAT3) pathways, which enhance cell survival, inhibit apoptosis, and increase growth capacity. Importantly, these pathways do not function in isolation but converge on a shared signaling network, with PI3K/Akt acting as a central hub frequently dysregulated in OC. Sustained activation of this axis promotes resistance to apoptosis and persistence of damaged cells, while MAPK/ERK signaling reinforces proliferative drive and contributes to cellular plasticity and motility (Ptak et al., 2014; Ptak et al., 2013; Ptak and Gregoraszczuk, 2012).

Beyond canonical signaling, emerging evidence indicates that BPA-driven ESR1 activation also reprograms cellular metabolism, thereby linking endocrine disruption to metabolic adaptation. BPA enhances glycolysis in OC cells through upregulation of key enzymes such as hexokinase 2 (HK2), resulting in increased lactate production (Shi et al., 2017). Mechanistically, this effect is mediated through activation of the estrogen receptor 1/phosphoinositide 3-kinase/protein kinase B/mechanistic target of rapamycin/hypoxia-inducible factor 1 alpha (ESR1/PI3K/Akt/mTOR/HIF-1α) axis, which promotes glucose uptake, ATP generation, and transcription of glycolysis-related genes. Notably, pharmacological inhibition of ESR1 attenuates these metabolic effects, supporting a receptor-dependent mechanism (Xie et al., 2023). This metabolic shift further induces histone H3 lysine 18 lactylation (H3K18la), which upregulates the RNA-binding protein IGF2BP3, stabilizes HK2 mRNA, and establishes a positive feedback loop that sustains glycolytic activity and tumor-promoting phenotypes (Xie et al., 2026). In addition to ESR1, BPA interacts with multiple nuclear receptors, including progesterone receptor (PGR) and glucocorticoid receptor (NR3C1) and mineralocorticoid receptor (NR3C2*)*, thereby amplifying downstream signaling outputs and further disrupting hormonal balance. Consistently, *in vivo* studies demonstrate that BPA exposure alters ERα/ERβ activity, disrupts steroidogenic processes (e.g., aromatase and StAR expression), and activates PI3K/Akt signaling in ovarian tissue, indicating broad interference with hormone-regulated networks (Bujnakova Mlynarcikova and Scsukova, 2024). BPA can also activate non-classical estrogen signaling through the G protein-coupled estrogen receptor 1 (GPER1; formerly known as GPR30), further expanding its signaling repertoire (Hoffmann et al., 2017a). Additionally, BPA and its halogenated derivatives have been shown to upregulate apelin via a peroxisome proliferator-activated receptor gamma (PPARγ) pathway, contributing to enhanced proliferation and migration (Hoffmann et al., 2017b).

At the tissue level, persistent activation of these interconnected pathways disrupts cellular homeostasis. Continuous stimulation of PI3K/Akt and MAPK/ERK, together with sustained transcription of cell cycle regulators, shifts cells toward a pro-proliferative state while weakening checkpoint control. BPA further facilitates this imbalance by suppressing tumor-inhibitory signaling, such as transforming growth factor-β (TGF-β) signaling, through upregulation of Ski-related novel protein N (SnoN) and inhibition of mothers against decapentaplegic homolog 3 (Smad3) phosphorylation (Park and Choi, 2014). Over time, these alterations promote clonal expansion of aberrant cells and support tumor initiation and progression. Importantly, similar receptor-binding and signaling effects have been observed for BPA analogues such as BPS, suggesting that similar hormone-receptor-related effects have also been reported for some BPA analogues, although the evidence should not be assumed to be directly interchangeable with that for BPA. *In vivo* exposure to BPS induces histopathological changes in ovarian and uterine tissues, along with transcriptional alterations in hormone metabolism and immunoinflammatory pathways (Yue et al., 2023). Complementary network-toxicology and Mendelian-randomization analyses have identified potential associations between BPA-related targets and OC–related phenotypes. These findings may help generate hypotheses regarding pathways such as PI3K/Akt, cell-cycle regulation, and stress responses; however, they do not establish direct pathway activation, causality, or mediation by these pathways (Shi et al., 2025).

Growing evidence indicates that BPA may affect OC-related biological processes primarily by dysregulating ESR1-centered hormone receptor signaling and its downstream networks, including PI3K/Akt and MAPK/ERK pathways, as well as metabolic and epigenetic programs, which may be associated with altered proliferative signaling and cellular homeostasis in selected experimental models.

### 4.2 Regulation of gene expression and transcriptional reprogramming

BPA exposure induces extensive transcriptional reprogramming that reshapes gene networks governing cell proliferation, apoptosis, metabolism, and inflammation (Ptak et al., 2011). Rather than causing isolated gene-level changes, BPA may induce coordinated alterations in interconnected regulatory modules, with hub genes such as lipocalin 2 (LCN2), lipase A (LIPA), and nuclear receptor subfamily three group C member 1 (NR3C1) linking endocrine disruption to metabolic and inflammatory signaling. Consistent with this network-level organization, transcriptomic analyses of ovarian cells exposed to estrogenic compounds, including BPA, have identified shared gene expression signatures enriched in ribosome biogenesis and RNA processing pathways. High-throughput sequencing studies further demonstrate that BPA induces coordinated changes in both mRNA and microRNA expression. RNA sequencing of normal human ovarian cells exposed to BPA identified differentially expressed genes that were enriched in pathways related to mRNA surveillance, cellular senescence, and transcriptional regulation. These enrichment results suggest candidate biological processes affected by BPA but do not demonstrate direct activation of these pathways or establish their causal involvement in ovarian carcinogenesis (Zahra et al., 2022). Notably, a subset of these BPA-responsive genes overlaps with those regulated by 17β-estradiol (E2), particularly in pathways associated with cell proliferation and migration, suggesting that BPA partially recapitulates estrogen-driven transcriptional programs (Márton et al., 2023). In line with this, BPA and other estrogenic compounds rapidly alter microRNA expression in an ERα-dependent manner, including members of the miR-200 family that are closely linked to epithelial identity and cell motility (Márton et al., 2020). Integration with TCGA datasets showed that some BPA-associated gene signatures, including HPDL, THY1, and PI3, were statistically associated with patient survival. These associations are exploratory and do not establish these genes as BPA-mediated prognostic biomarkers. These correlations provide potential clinical and prognostic clues, but they do not demonstrate that BPA regulates these genes in patients or that the identified genes mediate BPA-induced OC progression (Zahra et al., 2021). Moreover, these transcriptional changes were associated with distinct predicted immune-cell infiltration patterns in OC datasets. These findings suggest a possible relationship between BPA-associated gene signatures and tumor-microenvironmental features, but do not demonstrate direct immune remodeling by BPA (Yu et al., 2025). Additional integrative analyses identified hub genes, including CDKN1B, TIPARP, DYRK1B, EPCAM, and WDR77, within signatures associated with immune-related and tumor-progression pathways. These findings provide computationally derived hypotheses regarding BPA-related transcriptional networks but do not establish direct immune effects or validated prognostic biomarkers (Geng et al., 2026).

Functionally, this reprogramming converges on gene clusters associated with malignant phenotypes. RNA sequencing analyses in OC cells show that low-dose BPA exposure induces transcriptional programs associated with epithelial–mesenchymal transition (EMT) and activates canonical Wnt signaling, may be associated with enhanced invasive potential (Hui et al., 2018). Upregulation of matrix metallopeptidase 9 (MMP9) facilitates extracellular matrix degradation, while increased expression of C-X-C motif chemokine ligand 8 (CXCL8/IL-8) promotes a pro-inflammatory and pro-angiogenic microenvironment. Concurrent induction of prostaglandin-endoperoxide synthase 2 (PTGS2/COX-2) and tumor necrosis factor (TNF) further reinforces inflammatory signaling, forming a self-sustaining transcriptional circuit that stabilizes tumor-promoting programs (Hoffmann et al., 2018). In parallel, coordinated upregulation of cell cycle regulators such as CCND1 and MYC, together with anti-apoptotic factors including BCL-2 family members, shifts the balance toward sustained proliferation and survival (Shi et al., 2017).

A key feature of BPA-related transcriptional changes is its persistence, which is largely mediated by epigenetic remodeling. BPA modulates the expression of histone-modifying enzymes such as SET domain containing eight histone lysine methyltransferase (SET8) and sirtuin 1 (SIRT1) via estrogen receptor-dependent signaling, directly linking endocrine disruption to chromatin regulation (Hayes et al., 2016). In addition, BPA alters DNA methylation patterns through modulation of DNA methyltransferase (DNMT) activity and promotes activating histone modifications, including histone H3 lysine 27 acetylation (H3K27ac), via recruitment of p300/CBP. These epigenetic changes enable sustained activation of oncogenic loci beyond the initial exposure. BPA also reshapes non-coding RNA networks, including microRNAs and long non-coding RNAs, further fine-tuning gene expression at the post-transcriptional level. Mechanistically, BPA disrupts microRNA biogenesis through dysregulation of Exportin-5 and upregulates oncogenic microRNAs such as miR-21 via G protein-coupled estrogen receptor (GPER1)-dependent signaling (Lam et al., 2023). These alterations are associated with transcriptional signatures related to invasion, inflammation, and cell survival; however, their causal contribution to BPA-induced OC progression requires further experimental validation.

Accumulating evidence suggests that BPA may induce coordinated and potentially persistent changes in transcriptional regulation, involving both coding and non-coding genes as well as epigenetic modifications. These alterations may be associated with tumor-related phenotypes relevant to OC, although their contribution to OC progression remains uncertain.

### 4.3 Promotion of malignant cellular phenotypes

The cumulative outcome of BPA-associated signaling changes and transcriptional reprogramming is the establishment of malignant cellular phenotypes that may support tumor progression. Rather than acting through isolated pathways, BPA functionally integrates proliferative, survival, migratory, and stemness-related programs, resulting in a coordinated shift toward an aggressive cellular state. Consistently, in selected estrogen-responsive OC cell models, including BG-1 and OVCAR-3 cells, BPA has been reported to enhance proliferation, migration, or other tumor-related cellular phenotypes through estrogen-related signaling. However, these findings cannot be generalized to all OC because the histological and molecular characteristics of the models differ and were not always fully reported (Sang et al., 2021; Park et al., 2009). At the level of proliferation and survival, BPA sustains cell cycle progression while suppressing apoptotic responses. This is mediated by coordinated upregulation of key cell cycle regulators, including CCND1, CDK4/6, and MYC, which accelerate G1/S transition and may drive continuous cell division. In parallel, BPA enhances anti-apoptotic signaling through increased expression of B Cell lymphoma 2 (BCL-2) and B Cell lymphoma-extra-large (BCL-XL), coupled with suppression of pro-apoptotic mediators such as BCL-2-associated X protein (BAX) and caspases. This shift in apoptotic balance enables tumor cells to evade programmed cell death and maintain survival under stress conditions. Supporting this, *in vivo* studies demonstrate that BPA exposure disrupts apoptotic homeostasis in ovarian epithelial cells, characterized by increased BCL-2, decreased BAX, and activation of nuclear factor kappa B (NF-κB)-associated inflammatory signaling, changes that are associated with epithelial heterogeneity and early neoplastic transformation (Rajaura et al., 2024).

BPA also promotes invasive and migratory phenotypes by remodeling cell–matrix interactions and activating epithelial–mesenchymal transition (EMT) programs. Increased expression and activity of MMP2 and MMP9 facilitate extracellular matrix degradation and local invasion (Ptak et al., 2014). In parallel, BPA exposure has been reported to be associated with EMT-related changes in selected OC cell models, as evidenced by downregulation of E-cadherin (CDH1) and upregulation of mesenchymal markers such as N-cadherin (CDH2) and vimentin (VIM), along with activation of EMT-associated transcription factors including SNAI1, SNAI2, and TWIST1 (Kim et al., 2015). These changes enhance cellular plasticity and promote dissemination. Mechanistically, BPA also activates Wnt/β-catenin signaling, leading to upregulation of secreted phosphoprotein 1 (SPP1/OPN), which strengthens interactions between tumor cells and cancer-associated fibroblasts (CAFs), thereby facilitating stromal remodeling and metastatic spread. Disruption of this axis attenuates BPA-induced invasion and metastasis, highlighting its functional importance (Li and Li, 2026; Xu et al., 2025). In addition, remodeling of focal adhesion dynamics through FAK (PTK2) and Rho GTPases further enhances directional migration. Similar pro-invasive effects have also been reported for BPA analogues such as BPS and BPF, suggesting a conserved role of bisphenols in promoting invasive behavior (Gogola-Mruk et al., 2023).

In addition to promoting invasion, BPA may contribute to tumor progression by enhancing angiogenic capacity. BPA upregulates vascular endothelial growth factor A (VEGF-A) and its receptor VEGF-R2 in OC cells, thereby activating pro-angiogenic signaling pathways. Notably, this effect appears to differ from that of 17β-estradiol, which does not induce comparable VEGF expression changes, suggesting that BPA may exert distinct pro-angiogenic effects beyond classical estrogen signaling (Ptak and Gregoraszczuk, 2015). Another key feature of BPA exposure is the enrichment of stemness-associated tumor cell populations. BPA increases the proportion of CD44^+^ cancer stem–like cells *in vivo* and upregulates core stemness transcription factors, including NANOG, OCT4 (POU5F1), and SOX2 (Guo et al., 2020; Rajaura et al., 2024). Mechanistically, this effect has been linked to activation of a non-canonical PINK1/p53-dependent mitophagy pathway, which suppresses p53 nuclear translocation and may be relevant to tumor-related cellular phenotypes (Yuan et al., 2023). These stem-like cells exhibit enhanced tumor-initiating capacity, resistance to chemotherapy, and increased adaptability to microenvironmental stress through mechanisms such as drug efflux, cellular quiescence, and efficient DNA damage repair (Guo et al., 2020). At the systems level, these phenotypic changes are mutually reinforcing. These findings indicate tumor-related cellular changes in experimental models; however, their relevance to metastatic progression, disease aggressiveness, or clinical outcomes remains uncertain. Emerging evidence further suggests that BPA-induced oxidative stress may support the maintenance of cancer stem–like properties, indicating a functional link between redox imbalance and stemness regulation (Rajaura et al., 2024). Consistent with this, BPA has been shown to induce oxidative stress, apoptosis, and autophagy in ovarian granulosa cells through increased reactive oxygen species production, highlighting the broader impact of BPA on ovarian cellular homeostasis (Celar Sturm and Virant-Klun, 2023).

Existing evidence suggests that BPA is associated with several progression-related phenotypes in selected OC models, including increased proliferation, migration, invasion, angiogenesis-related changes, and cancer stem-like characteristics. However, these findings do not establish that BPA promotes metastatic spread or causes a globally aggressive tumor phenotype *in vivo*.

### 4.4 Oxidative stress and cellular damage responses

BPA exposure disrupts cellular redox homeostasis and has been linked to persistent oxidative stress, representing a central mechanistic link between environmental exposure and macromolecular damage in OC. At the cellular level, BPA promotes excessive production of reactive oxygen species (ROS) through mitochondrial dysfunction and activation of nicotinamide adenine dinucleotide phosphate oxidases (NOX enzymes). Impairment of the mitochondrial electron transport chain increases electron leakage and superoxide generation, while NOX activation further amplifies intracellular ROS accumulation. This pro-oxidant state is reinforced by suppression of antioxidant defense systems, including reduced activity of superoxide dismutase (SOD), catalase (CAT), and glutathione peroxidase (GPx). Consistently, *in vitro* studies show that BPA and its analogues elevate ROS levels while reducing SOD activity, accompanied by increased secretion of pro-inflammatory cytokines (IL-6, IL-8) and pro-angiogenic factors such as VEGFA, linking oxidative stress to a tumor-supportive microenvironment (Bujnakova Mlynarcikova and Scsukova, 2024). *In vivo* evidence further demonstrates increased ROS levels and downregulation of antioxidant genes including SOD1, SOD2, CAT, GPX1, and FOXO3 following BPA exposure (Rajaura et al., 2024). Clinically, elevated urinary BPA levels in OC patients are associated with increased ROS production and decreased SOD activity (p < 0.001), supporting the presence of BPA-related oxidative imbalance in humans (Alsaeed et al., 2026).

A defining feature of BPA-induced oxidative stress is its persistence and maladaptive regulation. Chronic BPA exposure has been reported to increase reactive oxygen species (ROS) production while impairing antioxidant defense mechanisms in ovarian tissues, as reflected by decreased expression of antioxidant-related regulators, including nuclear factor erythroid 2-related factor 2 (Nrf2), superoxide dismutase (SOD), catalase (CAT), glutathione peroxidase 1 (GPX1), and forkhead box O3 (FOXO3). This disrupted redox homeostasis may allow damaged cells to survive under sustained stress conditions, thereby favoring the gradual accumulation of genetic alterations. BPA has been reported to affect mitotic processes, including spindle formation, centrosome duplication, and chromosome segregation. However, whether these effects result in genomic or chromosomal instability in OC models remains insufficiently established (Bhardwaj et al., 2025; Kim et al., 2019).

Collectively, these processes indicate that BPA-induced oxidative stress may create a damage-prone oncogenic context characterized by persistent ROS accumulation, weakened antioxidant defense, and disruption of mitotic fidelity. These findings indicate a potential link between BPA-induced cellular damage and tumor-related signaling, but they do not establish genomic instability or ovarian epithelial transformation as direct consequences of BPA exposure.

### 4.5 Immune and inflammatory dysregulation

Bioinformatic analyses have identified associations between BPA-related gene signatures and predicted immune-cell infiltration patterns in OC datasets. These findings suggest a possible relationship between BPA-associated transcriptional profiles and the tumor microenvironment; however, immune-cell deconvolution estimates cell composition indirectly and cannot demonstrate that BPA directly alters immune-cell populations or immune function. Rather than acting solely as a pro-inflammatory stimulus, available evidence indicates that BPA may influence tumor–immune interactions through broader modulation of microenvironmental processes. Integrative transcriptomic and bioinformatic analyses provide initial evidence supporting this association. Immune infiltration analysis has revealed distinct immune cell profiles across BPA-associated gene expression groups, particularly involving genes such as HPDL, THY1, and PI3, suggesting a potential association with tumor-microenvironmental features. These gene networks are enriched in pathways related to cell cycle regulation, stress responses, and, in part, immune-related processes, suggesting that BPA-associated transcriptional changes may extend beyond classical oncogenic signaling to include modulation of tumor microenvironmental features (Yu et al., 2025).

Beyond immune-cell composition, BPA may be indirectly related to tumor–stroma interactions based on changes in stromal-associated factors; however, direct evidence for BPA-induced stromal remodeling in OC remains limited. For example, BPA-induced upregulation of secreted phosphoprotein 1 (SPP1/OPN) has been shown to promote fibroblast-to-CAF transformation and contribute to microenvironmental remodeling. Given the established role of stromal components in shaping the tumor microenvironment, these findings suggest a potential indirect link between BPA exposure and immune-related features, although direct evidence for immune modulation in this context remains limited (Xu et al., 2025). Furthermore, BPA-induced metabolic reprogramming, including enhanced glycolysis and activation of HIF-1α–associated signaling, may contribute to alterations in the tumor microenvironment. Given that metabolic states such as increased glycolysis and lactate accumulation are known to influence immune cell function, these findings suggest a potential link between BPA exposure and tumor–immune interactions (Shi et al., 2017; Xie et al., 2023; Xie et al., 2026). However, direct evidence connecting BPA-induced metabolic changes to immune modulation in OC remains limited.

**FIGURE 2:**
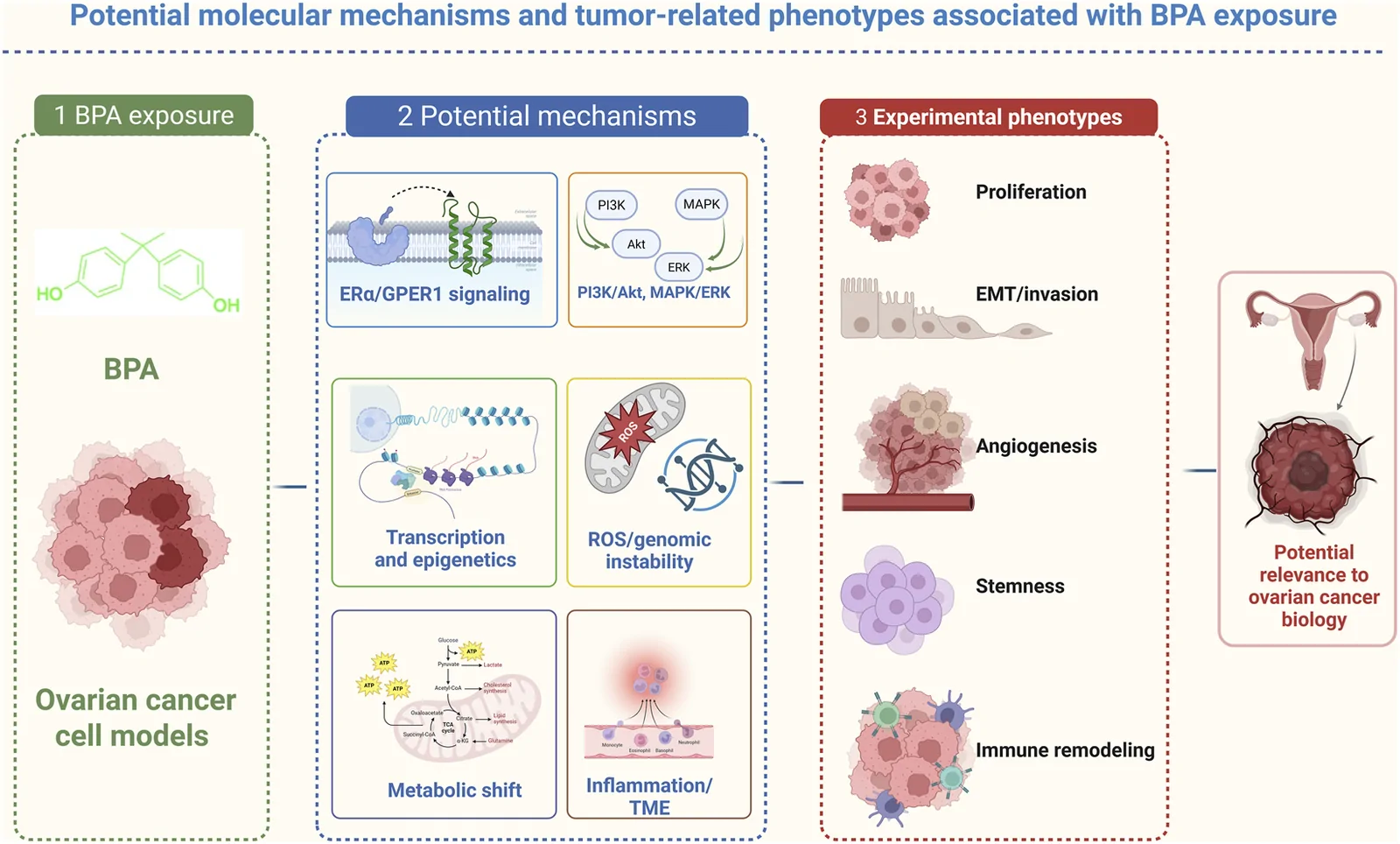
Schematic overview of potential mechanisms associated with BPA exposure and OC related phenotypes. The proposed mechanisms include estrogen receptor (ER) signaling, phosphoinositide 3-kinase/protein kinase B (PI3K/Akt) and mitogen-activated protein kinase/extracellular signal-regulated kinase (MAPK/ERK) pathways, transcriptional and epigenetic regulation, oxidative stress and genomic instability, metabolic alterations, and inflammation within the tumor microenvironment. The listed phenotypes, including proliferation, epithelial–mesenchymal transition (EMT)/invasion, angiogenesis, stemness, and immune remodeling, represent findings reported mainly in selected cellular or animal models. The right panel illustrates the potential relevance of these findings to OC biology rather than established clinical disease progression. The mechanisms shown are proposed or inferred from experimental and indirect evidence, and the connections between modules are schematic; they do not imply a confirmed causal sequence or establish causality in humans. Created with BioRender.com.

Current evidence provides preliminary and partly indirect support for an association between BPA-related molecular signatures and immune or microenvironmental features in OC. Direct experimental evidence demonstrating BPA-induced immune-cell remodeling in OC remains limited. These findings suggest a possible association between BPA-related molecular signatures and the tumor immune landscape. However, direct experimental evidence for BPA-induced immune-cell remodeling in OC remains limited, and the underlying mechanisms require further validation (Figure 2).

## 5 Conclusion and future outlook

This review summarizes epidemiological, cellular, animal, and integrative evidence concerning the possible relationship between BPA exposure and OC. Although some epidemiological studies have reported associations between higher urinary BPA levels and OC, these findings are limited by predominantly cross-sectional or case–control designs and cannot establish temporality or causality. In addition, not all studies have reported consistent associations, and urinary BPA measurements may be influenced by short biological half-life, exposure variability, and reverse causation. Experimental studies have shown that BPA can affect selected cellular outcomes, including proliferation, migration, oxidative stress, and endocrine signaling; however, these effects are not consistently observed across models and exposure concentrations. Findings based on pathway enrichment, molecular docking, transcriptomic correlations, or other indirect analyses remain hypothesis-generating and should not be interpreted as demonstrated mechanisms.

Several limitations restrict the interpretation of the current evidence. Most mechanistic studies use a limited number of established cell lines or animal models and may not adequately represent chronic, low-dose human exposure. Moreover, OC comprises distinct histological and molecular subtypes, whereas BPA responses have been investigated mainly in a small number of commonly used cell lines. Consequently, the reported effects may depend on the receptor status, genetic background, and experimental conditions of individual models. Evidence for genomic instability, immune remodeling, metastatic spread, chemotherapy resistance, or clinical biomarkers remains insufficient unless these outcomes have been directly measured in appropriate OC models or patient cohorts.

Future studies should prioritize prospective designs with repeated pre-diagnostic biospecimen collection to clarify exposure timing, temporal relationships, and potential dose–response patterns. Experimental research should use well-authenticated, physiologically relevant, and subtype-specific OC models, including models representing high-grade serous and other major subtypes. Integration of chronic low-dose exposure paradigms with clinically relevant molecular and functional endpoints may help determine whether the observed cellular effects are reproducible and biologically meaningful. At present, the available evidence supports biological plausibility but remains insufficient to establish that BPA causes OC or to validate BPA-related biomarkers for risk prediction, prognosis, or treatment.
